# Influence of demographic factors on the occurrence of motion artefacts in HR-pQCT

**DOI:** 10.1007/s11657-023-01352-5

**Published:** 2023-11-27

**Authors:** Stefan Benedikt, Lukas Rieser, Gernot Schmidle, Kerstin Stock, Lukas Horling, Gerald Degenhart, Rohit Arora

**Affiliations:** 1 Department of Orthopaedics and Traumatology, Medical University Innsbruck, Anichstraße 35, 6020 Innsbruck, Austria; 2 Department of Orthopaedics and Traumatology, Bezirkskrankenhaus Schwaz, Swarovskistraße 1/3, 6130 Schwaz, Austria; 3 Department of Radiology, Medical University Innsbruck, Anichstraße 35, 6020 Innsbruck, Austria

**Keywords:** HR-pQCT, Motion artefacts, Image quality, Age, Epidemiology, Demographic

## Abstract

***Summary*:**

The study shows a high incidence of motion artefacts in a central European population and a significant increase of those artefacts with higher age. These findings may impact on the design and conduct of future in vivo HR-pQCT studies or at least help to estimate the potential number of drop outs due to unusable image quality.

**Purpose:**

Motion artefacts in high-resolution peripheral quantitative computed tomography (HR-pQCT) are challenging, as they introduce error into the resulting measurement data. The aim of this study was to assess the general occurrence of motion artefacts in healthy distal radius and to evaluate the influence of demographic factors.

**Methods:**

The retrospective study is based on 525 distal radius second-generation HR-pQCT scans of 95 patients. All stacks were evaluated by two experienced observers and graded according to the visual grading scale recommended by the manufacturer, ranging from grade 1 (no visible motion artefacts) to grade 5 (severe motion artefacts). Correlations between demographic factors and image quality were evaluated using a linear mixed effects model analysis.

**Results:**

The average visual grading was 2.7 (SD ± 0.7). Age and severity of motion artefacts significantly correlated (*p* = 0.026). Patients aged 65 years or above had an average image quality between grades 1 and 3 in 72.7% of cases, while patients younger than 65 had an average image quality between grades 1 and 3 in 91.9% of cases. Gender, smoking behaviour, and handedness had no significant influence on motion artefacts.

**Conclusion:**

This study showed a high incidence of motion artefacts in a representative central European population, but also a significant increase of motion artefacts with higher age. This could impact further study designs by planning for a sufficiently large and if possible a more selective study population to gain a representative amount of high-quality image data.

## Introduction

High-resolution peripheral quantitative computed tomography (HR-pQCT) represents an innovative in vivo, non-invasive option for assessing volumetric bone mineral density and bone microarchitecture in the peripheral extremities. Until its introduction in 2005, numerous technical innovations and publications about the HR-pQCT emerged [1]. The device is particularly successful in the scientific field of bone mineral research, e.g. assessment of bone mineral density of the extremities, fracture detection and evaluation of fracture healing, monitoring of anti-osteoporotic therapies, assessment of secondary osteoporosis (including diabetes, renal diseases, rheumatic diseases, iatrogenic causes), and monitoring of inflammatory joint disorders [2]. HR-pQCT has the best signal-to-noise ratio and with 61 µm the highest spatial resolution of in vivo diagnostics. At the same time, radiation exposure for the patients is low with an effective radiation dose of 5 µSV per Stack [1, 3–5].

A disadvantage of this device is the relatively long scan time compared to other imaging methods, which predisposes the scans to significant motion artefacts [6]. Other imaging technologies as the cone-beam CT offer larger scan lengths and a shorter scanning time; however, resolution is lower and therefore susceptible for partial volume effects in thin trabecular structures [1]. Motion artefacts are challenging, as they have a significant impact on the image quality of the scans, thus making data difficult to process and assess and consequently introducing error into the resulting measurement data, particularly for trabecular and cortical microarchitecture [1]. Movement artefacts manifest as horizontal streaks, cortical smearing, and/or cortical interruptions within the images [7]. To determine the severity of motion artefacts, several grading systems have been developed, where the most commonly used is a 5 grade visual grading scale (grade 1 for no visible motion artefacts, grade 5 for major motion artefacts) [1]. The decision which images are acceptable for further assessment should be based on the research question [6]. Scans with a motion score of four may be acceptable for density-based measurements but are not recommended for trabecular and cortical microarchitecture or biomechanical examinations. Scans with a visual grading of 5 should not be used [1].

However, even with a standardised scoring system, motion grading remains subjective, and inter- and intra-observer agreement has shown to remain only moderate, even with an experienced observer [6, 8–10]. Automated techniques analysing HR-pQCT scans might provide a more standardised and objective approach [9, 11]; however, the best possible image quality should always be the main priority.

Several strategies for reducing motion artefacts were recently described. A standardised and reproducible study protocol and a well-trained and well-organised team form the basis for reliable results [10]. The limbs should always be immobilised in the manufacturer’s motion restraining holder [1]. Forearm casts with an additional thumb up part in fracture immobilisation [8] as well as repeated scanning [6, 9] showed a higher image quality. The selected scan area also correlates with the severity of motion artefacts [12].

Data regarding the occurrence of motion artefacts especially in combination with demographic factors are sparse. Regarding the fact that images with visual grading of 4 or 5 are mostly not recommended for further evaluation, as they lead to severe falsification of measurement data [6, 10], the consideration of possible dropouts due to poor image quality is essential when planning a study. Paggiosi et al. [13] described increasing age in correlation with more severe motion artefacts in a first-generation HR-pQCT. Bevers et al. [8] showed that age significantly correlates with more severe motion artefacts in scaphoid scans of the wrist when using a second-generation HR-pQCT. Such data are still missing for radius scans of the 2nd-generation HR-pQCT. Especially for studies on certain demographic groups (e.g. elderly osteoporotic female patients, young athletes, heavy workers), the knowledge about potential demographic influences on image quality could help to better plan the necessary inclusion number or at least help to estimate the potential dropout rate due to motion artefacts.

Therefore, the aim of this study was to assess the general occurrence of motion artefacts in healthy distal radius and to evaluate the influence of demographic factors on image quality using a second-generation HR-pQCT.

## Patients and methods

### Study design and population

The current retrospective study is based on distal radius HR-pQCT scans of a previously conducted prospective clinical study of our department. For the current study, data of 95 patients with unilateral conservatively treated traumatic distal radius fractures (aged > 18 years) presenting between December 2016 and December 2019 at our department were included [14]. All patients received up to six scans of their fractured and contralateral non-fractured wrist 1, 3, 5, 12 weeks, 6 and 12 months after trauma. For the current study, only the contralateral, non-fractured side was included. Patients were excluded in case of pre-existing conditions that affect the musculoskeletal system in any form as well as in case of pregnancy because of the potential teratogenic radiation dose.

The study was performed according to the ethical standards defined by the Declaration of Helsinki (revised in 2013). The study was approved by the institutional review board of the Medical University Innsbruck (No. AN2014-0374) and all patients gave their written consent for participating in this project.

### Demographic data acquisition

Demographic data were collected by questionnaire and included age, gender, smoking behaviour (smoker, non-smoker), handedness (left, right, ambidextrous), and profession (student, blue-collar worker, white-collar worker, retired).

### Scan acquisition

**Fig. 1:**
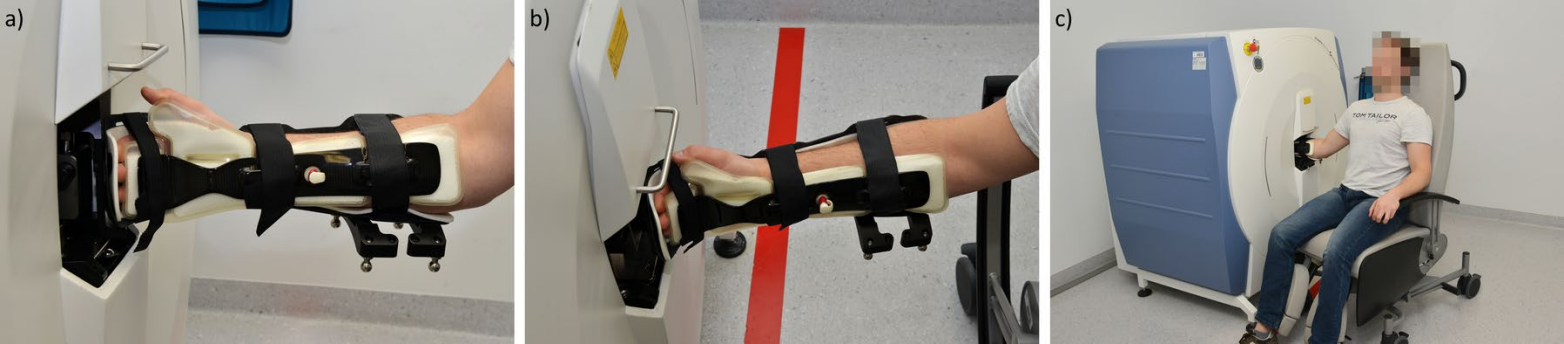
**a**–**c** Patient positioned in the appropriate seat provided by the manufacturer with the wrist immobilised in a thumb up position in the appropriate motion-restraining holders with inflatable pads

**Fig. 2:**
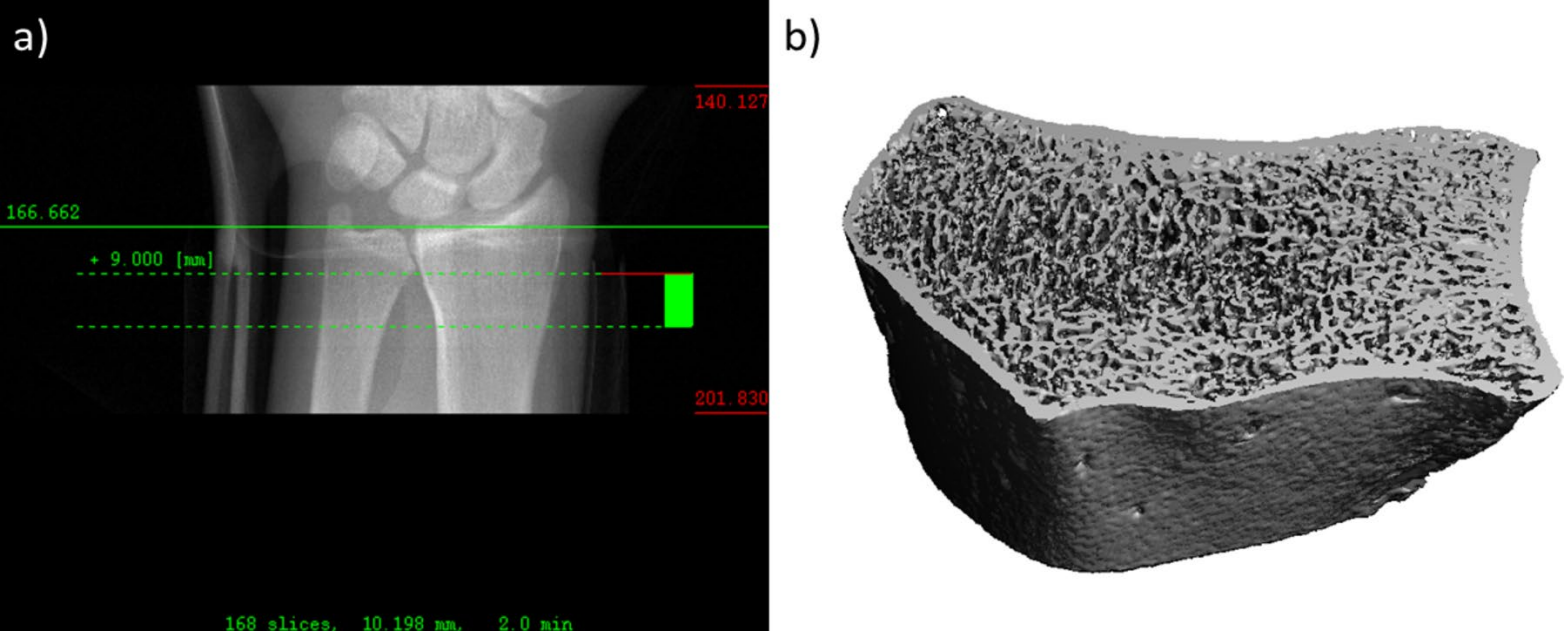
**a** Scout view of a right wrist with the reference line placed at the level of the radiocarpal joint space between the radius and the lunate. The region of interest was defined by a 10.2-mm wide area, 9-mm proximal to the reference line. **b** 3D reconstruction of the region of interest

All scans were performed using a second-generation HR-pQCT (XtremeCTII, Scanco Medical, Switzerland). To reduce the occurrence of motion artefacts, the patients’ wrists were immobilised in a thumb up position in the appropriate motion-restraining holders with inflatable pads provided by the manufacturer [1] (Fig. 1a–c). The healthy, non-fractured radius was visualised in one stack. The reference line was placed in the scout view conform standard protocol to determine the scan region (Fig. 2a and b). Pre-settings were a resolution of 60.7-µm isovoxels resulting in 168 slices per stack, 46-ms integration time, 68-kV voltage, and 1460-µA intensity. The scanning time per stack was approximately 2 min. The resulting radiation dose was approximately 5 µSV per stack. In order to monitor the longitudinal stability of the HR-pQCT system, scanning of the manufacturer’s quality control phantom containing rods of hydroxyapatite was performed daily. Repeated scanning in case of severe motion artefacts was not performed as it was not foreseen in the previous study from which the image data were obtained.

For post-processing, the provided Scanco medical software package was used (including multiprocessing virtual memory-based operating system VMS (©Hewlett-Packard, Palo Alto, USA) and image processing language IPL (Image Processing Language, Scanco Medical AG, Bruttisellen, Switzerland)).

### Classification of motion artefacts

**Fig. 3:**
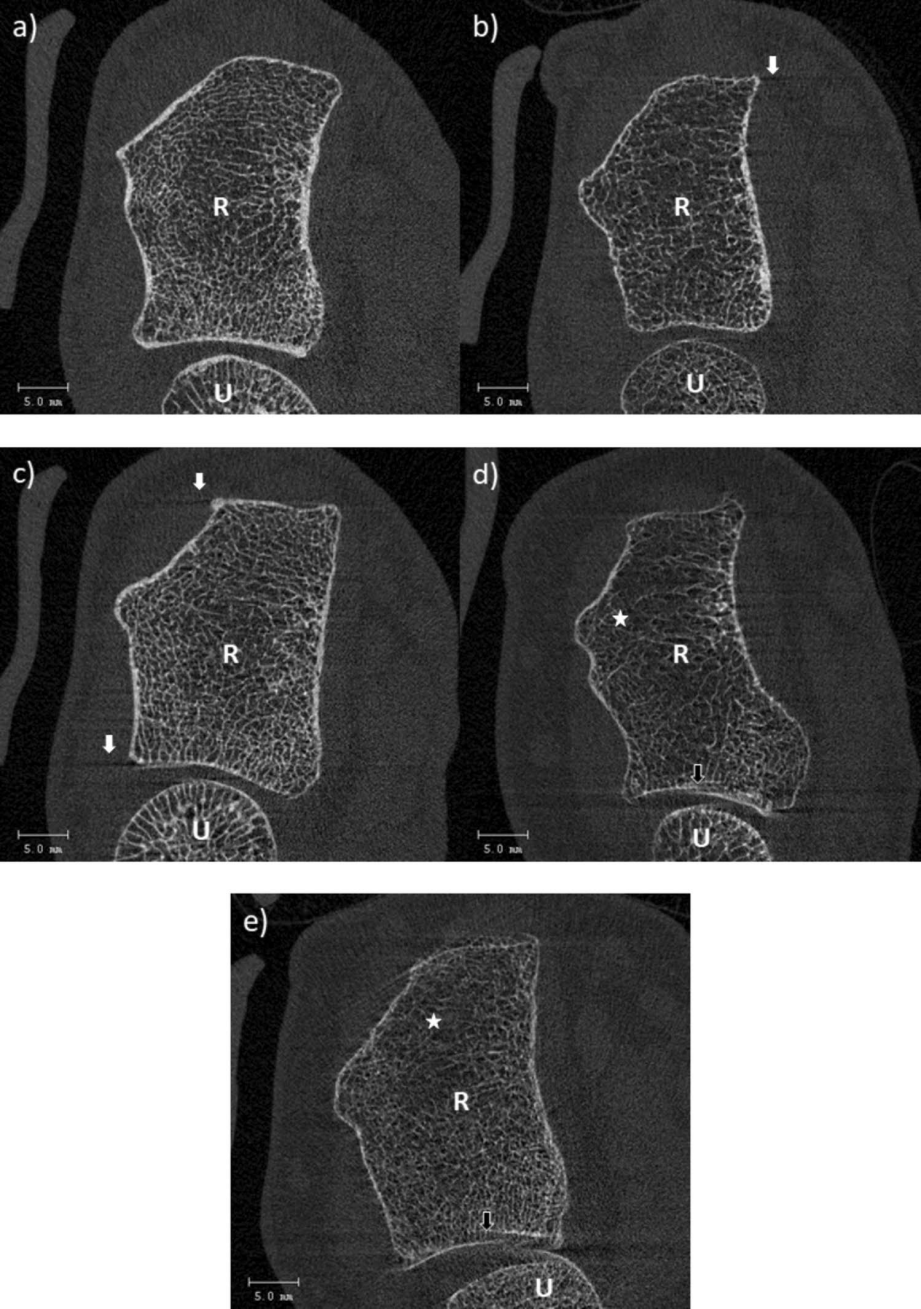
Visual grading scale of the distal radius. **a** Grade 1, no visible motion artefacts. **b** Grade 2, slight horizontal streaks (white arrow). **c** Grade 3, prominent horizontal streaks are visible, but the cortex is intact (white arrows). **d** Grade 4, prominent horizontal streaks, minor disruptions of the cortex continuity (black arrow), and minor trabeculae smearing (white asterix). **e** Grade 5, prominent horizontal streaking, considerable disruption of the cortical continuity (black arrow), considerable trabecular smearing (white asterix). R, radius; U, ulna

Images were assessed with the provided Scanco Medical evaluation software. All stacks were evaluated by two experienced observers (2nd and 4th author) separately from each other to reduce subjectivity in grading and to receive reproducible data. The motion artefacts were classified according to the visual grading scale described by Sode et al. [6, 7] and suggested by the manufacturer (Fig. 3a–e). Stacks were assessed slide by slide and image quality was defined by the most severe occurring motion artefact [14].

### Statistical analysis

Statistical analysis was performed using SPSS (IBM Corp. Released 2019. IBM SPSS Statistics for Windows, Version 26.0, Armonk, NY, USA). The collected data were first analysed descriptively using mean and standard deviation. The correlations between image quality and demographic factors were evaluated using a linear mixed effects model analysis. This statistical test takes into account the correlation between repeated measurements within the same subject in repeated longitudinal measures. Age, smoking behaviour, handedness, and gender were selected as fixed effects, while the time points of the follow-up (one to six follow-ups per patient) were selected as random effect. *P*-values < 0.05 were considered significant. Age and image quality were finally visualised using a cross table. Patients were therefore separated into two groups with one group younger than 65 years and the other group aged 65 years or above. The cut off at the age of 65 was chosen first as this is a common boundary for defining elderly individuals in medicine [15] and second as retirement age in the present country is around 65 years.

## Results

A total of 525 scans of 95 patients were analysed. Patient characteristics are shown in Table 1.

**Table 1:** Patients’ characteristics with the absolute number (*n*) and the relative number in percent

Demographic data (*n* = 95)	
Age	52.7 years (SD ± 19.2); range 18–91 years
Gender	Female *n* = 65 (68.4%)
	Male *n* = 30 (31.6%)
Handedness	Right-handed *n* = 87 (91.6%)
	Left-handed *n* = 2 (2.1%)
	Ambidextrous *n* = 6 (6.3%)
Dominant wrist scanned	*n* = 56 (58.9%)
Non-dominant wrist scanned	*n* = 39 (41.1%)
Smoking behaviour	Smoker *n* = 24 (25.3%)
	Non-smoker *n* = 71 (74.7%)
Profession	Student *n* = 8 (8.4%)
	White-collar worker *n* = 40 (42.1%)
	Blue-collar worker *n* = 9 (9.5%)
	Retired *n* = 36 (37.9%)
	Not stated *n* = 2 (2.1%)

Average scan number per patient was 5.5 (SD ± 1.0) and ranged between one and six follow-ups. The average visual grading among all 525 scans was 2.7 (SD ± 0.7). Fifty-three scans achieved a perfect grade 1 image quality (10.1%). Grades 2, 3, 4, and 5 were observed in 143 (27.2%), 171 (32.6%), 119 (22.7%), and 39 (7.4%) cases, respectively.

**Fig. 4:**
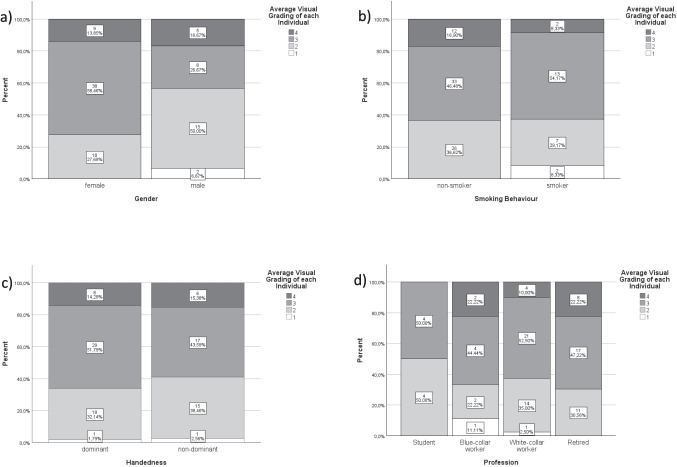
**a**–**d** Percentage distribution of the patients’ average visual gradings divided among gender, smoking behaviour, handedness, and profession. The white boxes contain the respective absolute and the relative results

Patients aged 65 years or above had an average image quality between grades 1 and 3 in 72.7% of cases, while 27.3% had grade 4 or 5. Patients younger than 65 had an average image quality between grades 1 and 3 in 91.9% of cases, while 8.1% had grade 4 or 5 (Table 2). The mixed model analysis revealed a significant correlation between age and the severity of motion artefacts (*p* = 0.026) (Table 3). The distribution of motion artefacts regarding gender, smoking behaviour, and handedness is depicted in Fig. 4a–c. No significant correlation was found between severity of motion artefacts and these factors (Table 3). Students achieved the best visual gradings followed by white-collar workers and blue-collar workers. Retired individuals had the most severe motion artefacts (Fig. 4d). To avoid multicollinearity errors, profession was excluded from the linear mixed effects model analysis, as there is a strong correlation between age and profession.

**Table 2:** Cross table visualising motion artefacts among age separated into two groups (< 65 years and ≥ 65 years). Since only two patients had an average image quality (among the six scans) of one and no patient had an average image quality of five, visual gradings 1 + 2 and 4 + 5 were grouped together

			Average visual grading of each patient			
			1 + 2	3	4 + 5	total
Age	< 65	Number	25	32	5	62
		In percent	40.3%	51.6%	8.1%	100.0%
	≥ 65	Number	10	14	9	33
		In percent	30.3%	42.4%	27.3%	100.0%
Total		Number	35	46	14	95
		In percent	36.8%	48.4%	14.7%	100.0%

**Table 3:** Results of the linear mixed effects model analysis comparing image quality (visual gradings 1–5) to demographic factors (gender, handedness, smoking behaviour, and age). Dependent variable was the mean visual grading between both observers

Source	*F*	Sig
Intercept	292.592	< 0.001*
Gender (male/female)	3.791	0.052
Handedness (dominant/non-dominant)	0.003	0.954
Smoker (yes/no)	1.352	0.246
Age per year	4.968	0.026*

*Significant (*p* < 0.05)

## Discussion

This study showed a considerable incidence of motion artefacts in a representative central European population and a significant influence of age on the severity of motion artefacts in in vivo HR-pQCT scans of the distal radius. The higher the age, the higher the probability for poor image quality. In individuals aged 65 years or above, the percentage of a visual grading ≥ 4 was 27.3%, meaning that at least nearly one-fourth of the images are not recommended for further microarchitectural evaluation.

The higher rate of motion artefacts in the elderly can be explained by common age-related movement disorders, for example essential tremor or by undiagnosed neurological disorders. Wenning et al. [16] found that in a randomly selected population of 706 cases from central Europe, the mean incidence for movement disorders of men and women was 18.5% in individuals aged 50–59 years and 51.3% in individuals aged 80–89 years. The prevalence of tremor was 14.5%, followed by restless legs syndrome in 10.8%, parkinsonism in 7%, and primary and secondary dystonia in 1.8%. A fifth of all movement disorders was diagnosed to be drug induced.

Pre-existing conditions with chronic pain may also limit the ability to stay still in one position in the above-mentioned chair and the motion-restraining holders, e.g. back pain that forces patients to change their sitting position (Fig. 1a–c). Cheung et al. [17] examined 1570 cases of a randomly selected population and found that individuals with chronic pain were on average significantly older. Joint (45.5%), muscle (21.7%), and back (25.2%) pain were the most common causes. Jakobsson et al. [18] showed in a randomised sample of 826 participants that up to 55% of people over 60 years of age suffered from chronic pain. However, it is unclear to what extent pain influences the development of motion artefacts in the HR-pQCT. In the present study, the study population was healthy, except of the contralateral distal radius fracture. However, while the presence of previous diseases was recorded in the medical history, possible false statements or undiagnosed disorders cannot be excluded with certainty.

Although male gender and smoker achieved better visual gradings compared to female individuals and non-smoker (Fig. 4a and b), no other demographic factors than age significantly correlated with the severity of motion artefacts.

Regarding the profession, retired persons had the worst outcome, which can be explained by the higher age compared to students and the working population. In contrast, students achieved better image quality.

Paggiosi et al. [13] in 2014 already analysed the influence of gender, examination site and age on the image quality of first-generation HR-pQCT scans of the radius and the tibia. Radius scans of male participants were more often classified as G1, than those of female participants (men 7–27%, women 10–20%). Images with slight or moderate movement G2 or G3 were observed in 73–87% in men and in 67–83% in women. Images classified as G4 were observed in 0–13% in men and in 10–16% in women. Repeated scans were more often indicated in patients aged over 70 years (16–18 years 9%, 30–32 years 3%, > 70 years 12.5%). For the tibia, the majority of scan images was graded as G1. Only 0–3% of the images were graded as G4 in both genders. Repeated scans were more often indicated in older individuals (16–18 years 0.8%, 30–32 years 0.8%, > 70 years 1.7%) [13].

In the current study, age significantly correlated with motion artefacts which confirms the results of Paggiosi et al. [13]. Male individuals also presented with less severe motion artefacts (Fig. 4a) compared to female individuals. However, the correlation between the severity of motion artefacts and gender was not significant in our study.

A detailed comparison between the two studies is difficult. Paggiosi et al. [13] used the grading system described by Engelke et al. [12] with only four categories ranging from G1 (perfect quality) to G4 (unacceptable movement artefacts), while the current study used the classification described by Sode et al. [7] ranging from grade 1 to grade 5. In the study by Paggiosi et al. [13], motion artefacts were assessed by only one single observer, which reduces the objectivity of the grading. Furthermore, scans were performed on the non-dominant limb except in cases of prior fractures and a maximum of one repeated scan at each site was carried out in case of unacceptable movement artefacts. In the current study, the healthy non-fractured wrist was scanned, and repeated scans were not performed. The impact of demographic factors on motion artefacts such as smoking behaviour, handedness, and profession was not examined in the study of Paggiosi et al. [13]. The study design and the patient collective also differ. Paggiosi et al. [13] performed a cross-sectional study and included 180 healthy participants, with 30 men and 30 women aged between 16 and 18, between 30 and 32, and over 70, respectively, while in the present retrospective study, healthy patients with a contralateral distal radius fracture over 18 years but apart from that regardless of gender were included. Paggiosi et al. [13] used the first-generation HR-pQCT, while in the current study, a second-generation device was used. The devices differ primarily in terms of their resolution (1st generation: 82 µm; 2nd generation: 61-µm voxel size), their number of slices per stack (1st generation: 110 images with a length of 9.02 mm; 2nd generation: 168 images with a length of 10.2 mm), and their integration time (1st generation: 100 ms; 2nd generation: 46 ms) [1]. The positioning of the reference line also slightly differed. In individuals between 16 and 18 years with still visible growth plates, the scan started 1 mm away from the proximal end of the growth plate. In all other participants, the reference line was placed on the notch of the articular surface of the distal radius and on the endplate of the distal tibia to define the position of the first slide (9.5 mm from the reference line for the radius and 22.5 mm from the reference line of the distal tibia) [13]. Consequently, visual grading might differ in the studies influenced by the different number of slices (the more slices, the higher the chance for motion) and the different scan region (different anatomic structure) as well as by the potentially better image quality in second-generation HR-pQCT scans.

However, although the comparison is difficult, both studies report a clear association between image quality and age in distal radius scans.

Engelke et al. [12] compared the occurrence of motion artefacts between the radius and the tibia in 320 scans using a 1st-generation HR-pQCT. Both regions were separated in ultradistal and distal regions whereby for the radius ultradistal scans were defined as a stack 9.5 mm proximal to the reference line and distal scans as stacks 20 mm proximal to the endpoint of the ultradistal stack. They found that the ultradistal scans of the radius were more severely affected by movement artefacts than the other locations.

Pialat et al. [6] performed a retrospective study to investigate the occurrence of motion artefacts in the distal radius and distal tibia and the influence of these artefacts on bone density and microarchitecture. Image quality according to the manufacturers provided visual grading scale (grades 1–5) of non-repeated radius scans was in 39% of cases grade 1, in 38% grade 2, in 19% grade 3, in 3% grade 4, and in 1% grade 5. In the current study, the average image quality per patient was worse with 35% grade 1 or 2, 48.4% grade 3, and 14.7% grade 4 or 5 images. The average patient age in the study of Pialat et al. was 45.2–65.7 years (in the current study 52.7 years). However, the two studies are only comparable to a limited extent, mainly since on the one hand a first-generation HR-pQCT was used with the limitations mentioned above and on the other hand, the data set consisted of 4 different studies and not only of healthy individuals. Furthermore, the visual grading was based on only three slices (the distal, middle, and proximal slices of the 110-slice stack) while the current study evaluated each stack slide by slide and defined the quality grade according to the most severe occurring motion artefacts.

Bevers et al. [8] examined the feasibility of HR-pQCT in patients with suspected scaphoid fractures. In this study, they could show that age significantly correlated with more severe motion artefacts. However, the scaphoid bone differs from other bones in both its microarchitecture and its shape [19], has a poor interobserver and intraobserver reliability regarding visual grading of motion artefacts [10], and is rarely the target bone in HR-pQCT studies [2].

Strengths of this study include the high number of patients as well as the total number of 525 scans performed with up to six scans per patient. Moreover, the patient population covers an age range from 18 to 91 years. The image grading by two experienced observers contributed to more objective and reproducible results.

Beside the retrospective study design, a limitation of this study is the small number of subjects aged 65 years or above possibly impairing the robustness of the results, since motion artefacts occur more frequently in older patients [13]. The definition of the two age groups (< 65 and ≥ 65) itself might be a limitation, as improving life expectancy, quality of life, and level of function changes this traditional threshold.

The choice of the patient collective, which consisted exclusively of individuals with unilateral, contralateral radius fractures, might also have influenced the results: The general fracture pain as well as a possible pain-related difficile positioning in the examination chair and in the motion-restraining holders might have increased motion artefacts.

For the current study, only scans of the distal radius were available. An assessment of the impact of demographic factors on image quality of the tibia would also have been important as it is often the target bone in HR-pQCT studies.

Since only one stack was scanned at a time, no statements can be made about the artefacts to be expected in larger areas with a longer scan duration.

## Conclusion

In HR-pQCT studies, the best possible image quality has to be aimed for as it increases their scientific significance. However, motion artefacts can hardly be avoided in in vivo examinations. The results of this study show a considerable incidence of motion artefacts in a representative central European population, with a significant correlation between the severity of those motion artefacts and increasing age. Gender, handedness, and smoking behaviour had no significant influence. These findings may impact on the design and conduct of future in vivo HR-pQCT studies or at least help to estimate the potential number of dropouts due to unusable image quality. A rigorous, standardised, and reproducible study protocol, a well-coordinated team, adequate and comfortable positioning of the patient, a well-considered scan region, and repeated scanning in combination with a sufficiently large and eventually more selective study population if possible form the basis for high-quality image data.

## Data Availability

The data that support the findings of this study are available from the corresponding author upon reasonable request.
